# Enhanced antibacterial properties of amoxicillin-loaded silver nanoparticles against Methicillin-resistant *Staphylococcus aureus*: physicochemical characterization, anti-virulence activity, and biofilm inhibition

**DOI:** 10.7717/peerj.20924

**Published:** 2026-03-12

**Authors:** Naifa Alenazi, Reem Binsuwaidan, Samiah Alhabardi, Saud Suliman Alanazi, Reham M. Aldahasi, Jawza A. Almutairi, Wafa K. Fatani

**Affiliations:** 1 Department of Pharmaceutical Science, College of Pharmacy, Princess Nourah bint Abdulrahman University, Riyadh, Saudi Arabia; 2 Pharmaceutics Department, College of Pharmacy, King Saud University, Riyadh, Saudi Arabia; 3 Strategic Centre for Diabetes Research, College of Medicine, King Saud University, Riyadh, Saudi Arabia; 4 Department of Biology, College of Science, Princess Nourah bint Abdulrahman University, Riyadh, Saudi Arabia

**Keywords:** Methicillin-resistant *Staphylococcus aureus*, Silver nanoparticles, Amoxicillin, Antibacterial activity, Biofilm inhibition

## Abstract

Methicillin-resistant *Staphylococcus aureus* (MRSA) presents significant challenges in healthcare and community settings due to its diverse virulence factors and increasing resistance to conventional antibiotics. Given the scarcity of effective treatments, developing innovative antibacterial strategies is essential. This study explores the potential of silver nanoparticles conjugated with acacia extracts as nanocarriers for amoxicillin to enhance antibacterial efficacy and circumvent resistance mechanisms in MRSA. The synthesized amoxicillin-loaded silver-acacia nanoparticles were characterized for their physicochemical properties, revealing spherical morphology with a minimum particle size of approximately 230 nm, a polydispersity index of 0.3, and a high negative zeta potential of −32 mV as confirmed by transmission electron microscopy. *In vitro* assays demonstrated that these nanoparticles significantly inhibited bacterial growth, achieving a reduction at a minimum inhibitory concentration (MIC) of 2 mg/mL. At this concentration, biofilm formation by MRSA was inhibited by 80%, as verified by scanning electron microscopy, and hemolytic activity on blood agar was completely suppressed. While a dose-dependent cytotoxic effect on endothelial cells was observed, the MIC concentration remained cytocompatible (*p* < 0.05). These findings underscore the promise of amoxicillin-loaded silver-acacia nanoparticles as potent antibacterial agents with minimal cytotoxicity at effective doses. This study highlights the potential of nanotechnology-enabled drug delivery to repurpose amoxicillin and offers a novel platform for combating multidrug-resistant MRSA infections, which may inform future therapeutic developments.

## Introduction

Methicillin-resistant *Staphylococcus aureus* (MRSA) is a formidable pathogen responsible for a wide range of infections, including device-related infections, osteomyelitis, endocarditis, and skin and soft tissue infections. MRSA remains a significant public health concern in Saudi Arabia, with recent studies indicating prevalence rates ranging from 17% to 45% depending on region, population, and clinical setting. Higher rates are observed in hospitals (Alhejaili et al., 2025). The emergence of MRSA strains resistant to multiple antibiotics, including β-lactam agents, has significantly complicated treatment options, leading to increased morbidity and mortality rates worldwide. The rapid spread of MRSA is driven by factors such as increased transmission rates, colonization, and the activation of virulence factors that enhance pathogenicity (Alhejaili et al., 2025; Aljeldah, 2020). Silver nanoparticles (AgNPs) are emerging as highly promising antibacterial agents due to their broad-spectrum efficacy against both Gram-positive and Gram-negative bacteria, including multidrug-resistant strains (Rodrigues, Batista & Rodrigues, 2024; Simon et al., 2021). The mechanism of action involves the release of silver ions, which disrupt bacterial cell membranes, induce oxidative stress *via* reactive oxygen species, and interfere with essential cellular processes such as DNA replication and protein synthesis (Rodrigues, Batista & Rodrigues, 2024). Studies highlight AgNPs’ ability to enhance the effectiveness of existing antibiotics, making them valuable candidates for developing new antimicrobial formulations. Their unique physicochemical properties and synergy with other antibiotics position silver nanoparticles as a powerful tool in combating resistant pathogens in healthcare settings (Simon et al., 2021). AgNPs operate through various mechanisms of action, and when used in conjunction with antibacterial agents, such as organic compounds or antibiotics, they demonstrate a potent antibacterial effect against pathogenic bacteria, including *Escherichia coli* and *Staphylococcus aureus*. The unique properties of silver nanoparticles render them particularly advantageous for use in medical and healthcare applications, where they can effectively treat or prevent infections (Al Bshabshe et al., 2020). Acacia extracts exhibit potent antibacterial activity against a wide range of bacterial strains, including antibiotic-resistant ones. Studies show that *Acacia nilotica* extracts, particularly from leaves, pods, and bark, inhibit the growth of pathogens like *Escherichia coli, Salmonella*, and *S. aureus* by damaging bacterial cell membranes and affecting cell integrity. The antimicrobial effect is concentration-dependent, with higher concentration showing stronger inhibition zones. Acacia’s bioactive compounds offer promising potential as alternative or complementary antibacterial agents, especially in combating resistant bacterial strains (Simon et al., 2021).

This plant may serve as a promising source for the development of novel antimicrobials that are effective against strains of pathogens resistant to antibiotics (Aldahasi, Shami & Mohammed, 2024). Amoxicillin is a significant member of the beta-lactam antibiotic class, utilized in the treatment of various bacterial infections that are susceptible, particularly those affecting the skin, genitourinary system, as well as the ear, nose, and throat (Akhavan, Khanna & Vijhani, 2023). Amoxicillin is a widely used antibiotic classified as an essential medicine by the World Health Organization (WHO) due to its effectiveness against a broad range of bacterial infections, including pneumonia and ear infections. It belongs to the aminopenicillin class and is valued for its safety and accessibility worldwide. However, the growing issue of bacterial resistance limits its clinical utility, necessitating prudent use to preserve its efficacy (World Health Organization, 2025; Bruna et al., 2021). Resistance to amoxicillin underscores the urgent need for new methods to reuse this important medication, which would also be cost-effective. Developing innovative strategies to restore amoxicillin’s efficacy, such as utilizing antibiotic adjuvants, potentiators, or combination therapies, can help overcome resistance mechanisms and extend the lifespan of this essential drug. These approaches not only preserve amoxicillin’s clinical utility but also reduce healthcare costs by minimizing the necessity for more expensive alternative treatments.

The incorporation of silver nanoparticles (AgNPs) and Acacia extract presents a novel and robust antibacterial strategy, wherein AgNPs act as a protective carrier enhancing amoxicillin’s stability and potency at the infection site. This study uniquely investigates the effects of AgNPs conjugated with Acacia extract and amoxicillin (AgNPs-Amox) against MRSA. Comprehensive physicochemical characterization of the formulation was conducted, and its antibacterial, anti-virulence, and biofilm inhibitory properties were evaluated. The findings demonstrate the promising therapeutic potential of AgNPs-Amox as an innovative approach for effectively combating MRSA infections, addressing resistance and biofilm challenges.

## Materials and Methods

### Materials

Silver nitrate, ferrous chloride, ferric chloride, and potato-dextrose agar (PDA) were obtained from the Laboratory of Princess Nourah bint Abdulrahman University, Riyadh, Saudi Arabia. Bacterial strains, including MRSA (ST239-III) from American Type Culture Collection (ATCC), and *S. aureus* wildtype (S2014; Sigma-Aldrich, St. Louis, MO, USA). Agar media were purchased from Oxoid, UK. The Zetasizer Nano ZS90 (Malvern Instruments Ltd, UK) was used for particle size, polydispersity index (PDI), and zeta potential measurements. Transmission electron microscopy (TEM) was performed using a JEM1230EX instrument (Tokyo, Japan), scanning electron microscope (SEM) (FEI Company, Hillsboro, NC, USA). A microplate reader, Bioanalytics (Germany, France, UK, Switzerland). Acacia extract was obtained from Laboratory of Princess Nourah bint Abdulrahman University, Riyadh, Saudi Arabia. All tests conducted in triplicate.

### Preparation of AgNPs-Amox

Silver-acacia nanoparticles (AgNPs) were synthesized accordance to the previous study (Aldahasi, Shami & Mohammed, 2024). Combining 90 mL of 1 mM silver nitrate with 10 mL of Acacia extract, followed by heating at 90 °C for 15 min. The nanoparticles were centrifuged at 5,580 × g for 30 min, washed with distilled water, and dried. Amoxicillin (0.001 M) was added to the AgNPs, and was continuously stirred for 24 h at a temperature of 25 °C forming AgNPs-Amox (Sadiq et al., 2017).

### Characterization of nanoparticles

The particle size, PDI, and zeta potential of AgNPs-Amox were measured (water) using a Zetasizer. UV-Vis spectroscopy (Thermo Fisher Scientific, Waltham, MA, USA) was used solely for the quantitative determination of amoxicillin of 200–500 nm. Transmission electron microscope (TEM) was employed to assess the morphology and size of the nanoparticles. The encapsulation efficiency of silver nanoparticles loaded with amoxicillin was determined through the indirect spectrophotometric method. The nanoparticles underwent treatment with chloroform and were thoroughly shaken, followed by measurement spectrophotometrically at 228 nm. The encapsulation efficiency can be computed using the formula provided below:

Encapsulation Efficiency = Amount of Encapsulated Nanoparticle/Amount of Encapsulated Nanoparticle + Free Nanoparticle ×100.

### Antimicrobial activity assays

The minimum inhibitory concentration (MIC) of AgNPs-Amox was determined by growth assay. The growth of MRSA and *S. aureus* were assessed using optical density (OD) for 24 h at 600 nm wavelength. Different concentration were tested ranging from 0.6 to two mg/ml, to determine the lowest effective concentration. Vancomycin (two µg/mL) was used as positive control. The Kirby-Bauer disk diffusion method was used to evaluate the susceptibility of MRSA and *S. aureus* to AgNPs-Amox. Hemolysis was conducted using sheep blood agar plates to assess the anti-virulence capabilities of the nanoparticles. Both bacterial strains were suspended in buffer, standardized to turbidity (McFarland Standards 0.5).

### Biofilm assay

Biofilm assay was used to evaluate the effect of MIC of formulated nanoparticles on bacterial biofilm formation. Overnight bacterial cultures adjusted to 0.5 McFarland standard were inoculated (100 µL) into 96-well microtiter plates containing 100 µL of formulated nanoparticles and incubated at 37 °C with shaking at 150 rpm overnight. Following incubation, planktonic cells were removed, and wells were washed twice with 0.9% NaCl and air-dried for 1 h. Biofilms were stained with 0.1% crystal violet for 15 min, excess stain was removed by washing thrice with 0.9% NaCl, and bound dye was solubilized with ethanol-acetone (80:20 v/v). Absorbance was measured using an ELISA plate reader to quantify biofilm mass.

### Biofilm visualization *via* scanning electron microscopy

Biofilm formation was assessed using SEM. Briefly, an overnight culture of bacteria in Tryptic Soy Broth (TSB) was diluted to 10^7^ CFU/mL in Lysogeny Broth (LB). Aliquots (two mL) of the diluted culture were added to 6-well plates and two mL of fresh LB. Biofilms were allowed to form on the coverslips for 24 h at 37 °C. The resulting biofilms were fixed with 3% glutaraldehyde in phosphate buffer (pH 7.2) for 24 h, followed by post-fixation with 1% osmium tetroxide for 1 h. Dehydration was performed using a graded ethanol series (50–100%), after which the sample was dried, sputter-coated with a palladium-gold film, and imaged using SEM (Alfaraj et al., 2023).

### Cytotoxicity assessment *via* MTT assay

Human endothelial cells were cultured in Dulcecco’s Modified Eagle Medium (DMEM) supplemented with 10% (v/v) fetal bovine serum (FBS), 1% (v/v) penicillin, and 1% (v/v) streptomycin. The cells were maintained in a humidified incubator at 37 °C with 5% CO_2_. For cytotoxicity evaluation, cells were seeded at a density of 5  × 10^3^ cells per well in 96-well plates and allowed to adhere overnight under standard culture conditions. After adherence, the cells were treated with varying concentration of the AgNPs-Amox, ranging from 0.5 mg/mL to 4mg/mL, and incubated for 48 h at 37 °C with 5% CO_2_. penicillin/streptomycin antibiotics were removed from the culture medium during antimicrobial exposure to avoid confounding cytotoxicity results. Cell viability was assessed using the MTT assay.

### Statistical analysis

All experiments were performed in triplicate (*n* = 3 independent replicates). Statistical analysis was conducted using one-way ANOVA with a significance level set at *p* ≤ 0.05 for detecting meaningful differences. Following the ANOVA, we applied Tukey’s Honestly Significant Difference (HSD).

## Results

### Characterization of nanoparticles

**Figure 1:**
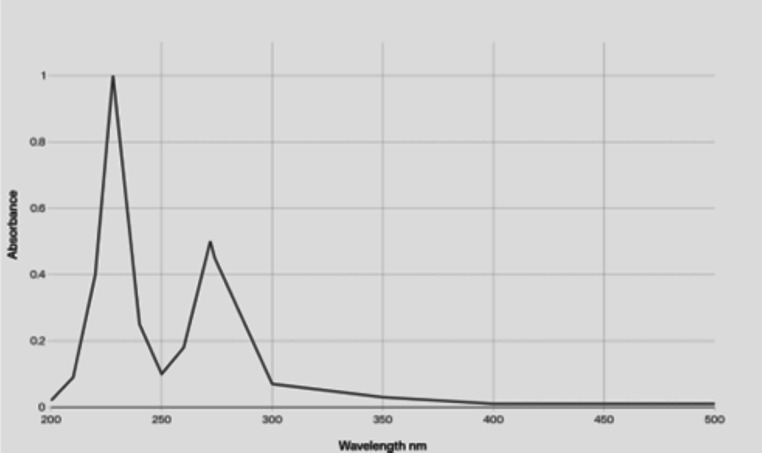
UV-Vis absorption spectrum for amoxicillin (0.001 M) in the range of 200–500 nm, showing characteristic absorption peaks at 228 nm and 274 nm.

**Figure 2:**
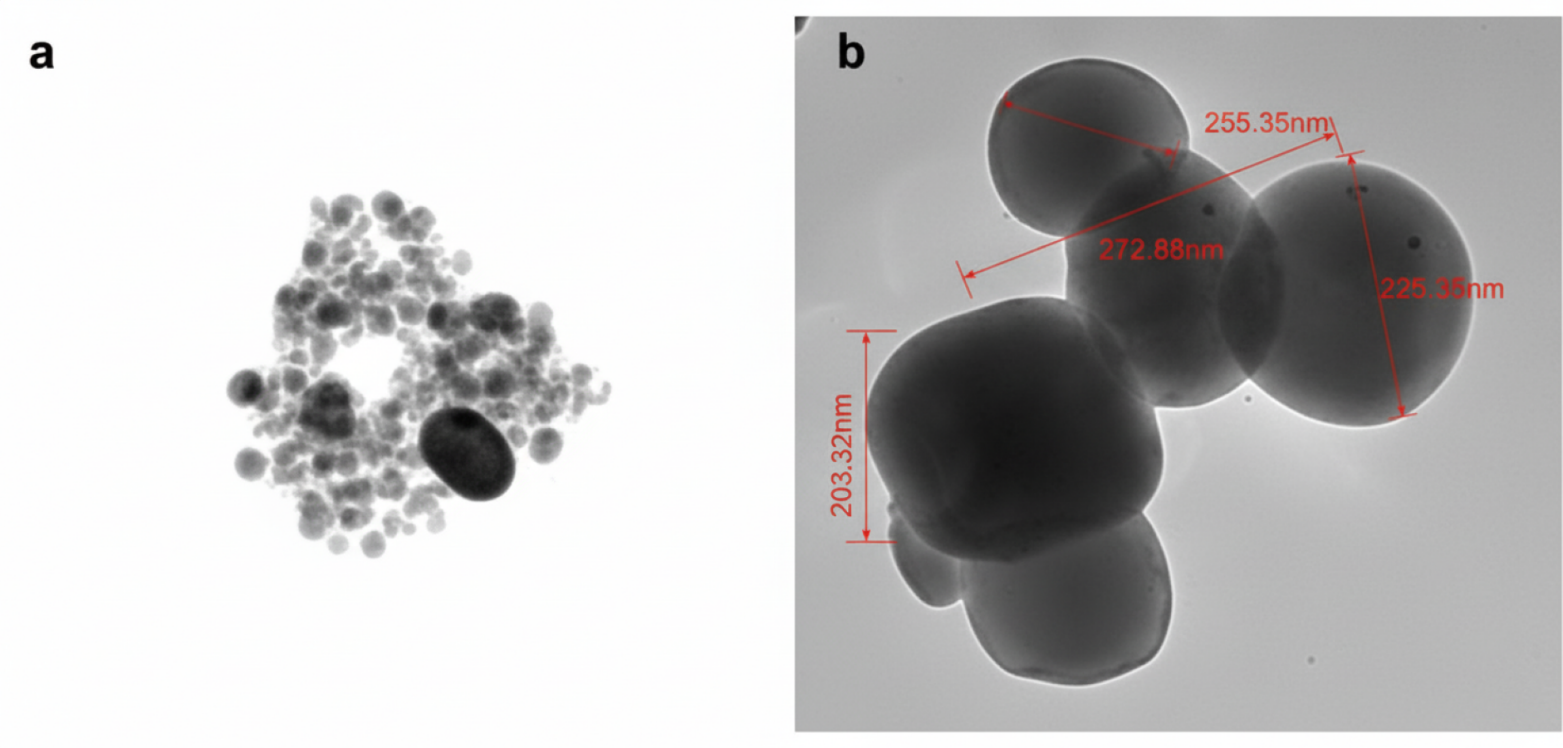
Transmission Electron Microscopy (TEM) images of nanoparticles. (A) Blank silver nanoparticles (AgNPs), (B) silver nanoparticles loaded with amoxicillin (AgNPs-Amox), demonstrating spherical morphology and size around 230 nm for drug-loaded nanoparticles.

**Figure 3:**
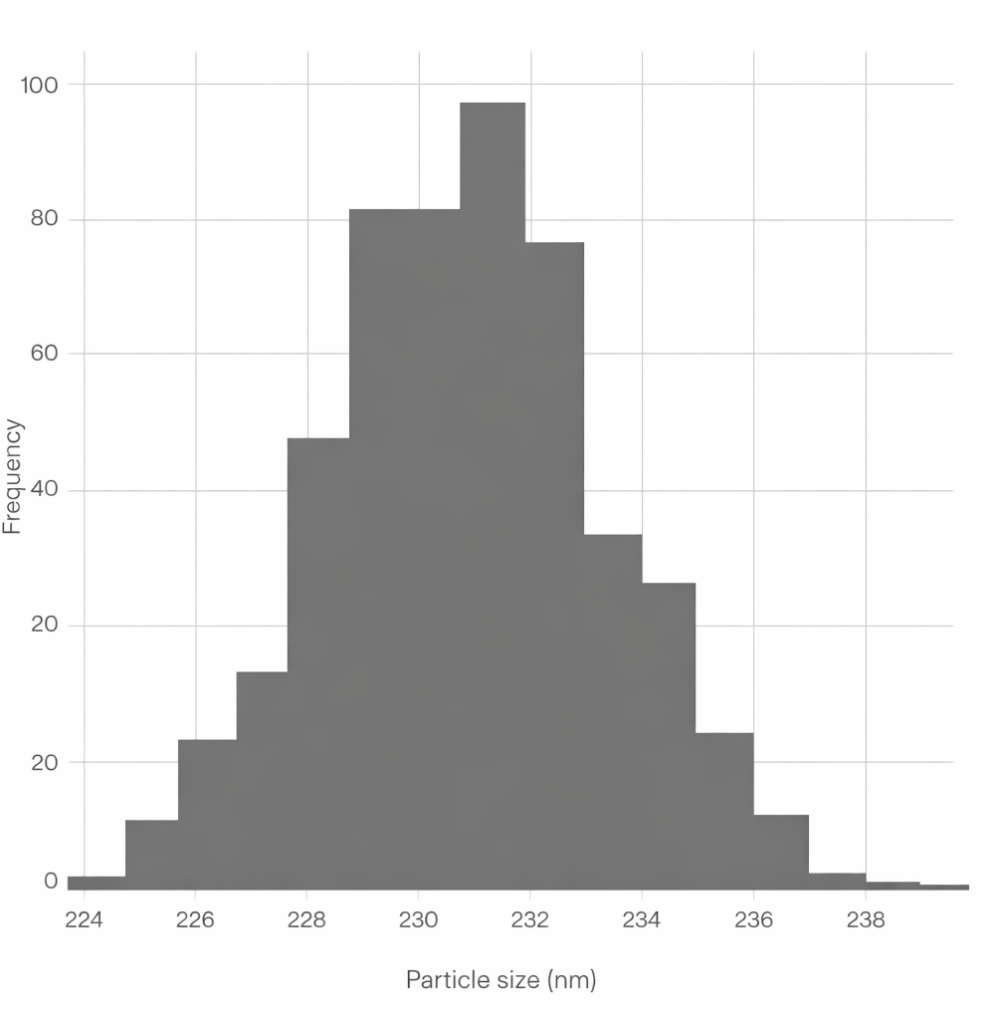
The histogram for silver-amox nanoparticles. Narrow, approximately normal size distribution centered around 230 nm.

The synthesized silver nanoparticles loaded with amoxicillin (AgNPs-Amox) were characterized for size, polydispersity index (PDI), and surface charge (zeta potential). Dynamic light scattering measurements revealed that AgNPs-Amox exhibited a mean particle diameter of 230 nm with a narrow size distribution indicated by a PDI of 0.31 (Guo & Li, 2023). The zeta potential was determined as −32 mV, suggesting good colloidal stability due to electrostatic repulsion between particles. In comparison, blank silver nanoparticles (AgNPs) showed a smaller average size of 120 nm, a lower PDI of 0.21, and a zeta potential of −24 mV (Table 1). The UV-Vis absorption spectrum for amoxicillin in the range of 200–500 nm, showing characteristic peaks at 228 nm and 274 nm (Fig. 1). Transmission electron microscopy (TEM) confirmed the spherical morphology of both nanoparticle formulations, with diameters consistent with size measurements (Fig. 2), and the histogram shows a narrow, and normal size distribution (Fig. 3). The encapsulation efficiency is defined as the percentage of drug that is entrapped successfully by the silver nanoparticles. Utilizing the formula provided:

Encapsulation Efficiency = Amount of Encapsulated Nanoparticle/Amount of Encapsulated Nanoparticle + Free Nanoparticle ×100.

It was determined that the quantity of encapsulated nanoparticles is 3.12 mg, while the total of encapsulated and free nanoparticles amounts to 4.2 mg. The encapsulation efficiency was computed to be 74.2%, which signifies a notably high and effective loading of silver amox nanoparticles.

### Antimicrobial activity

**Figure 4:**
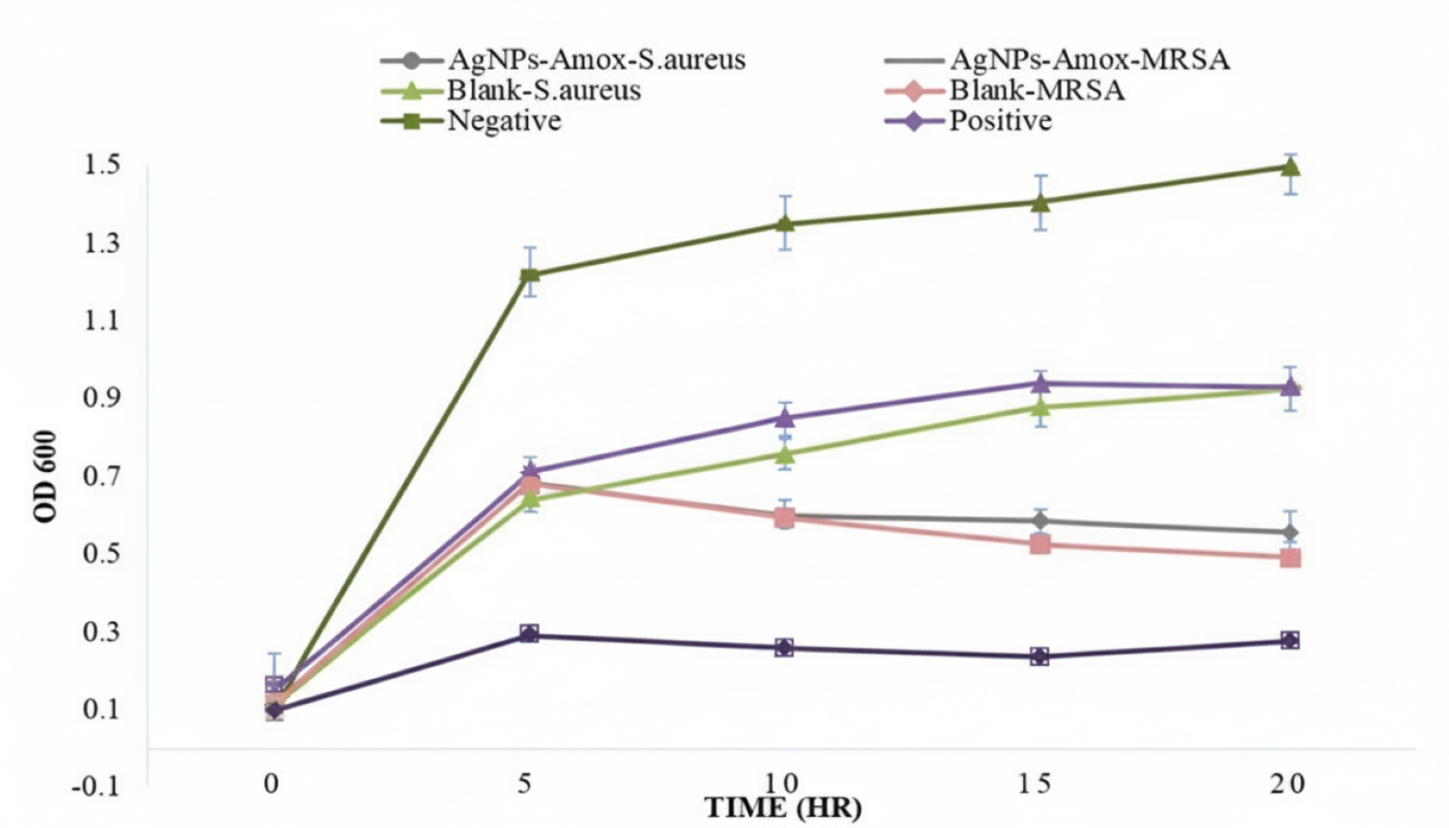
Growth curves of MRSA and *S. aureus* treated with formulated nanoparticles. Antimicrobial activity at MIC concentration of 2 mg/ml. Data presented as mean ± SD, *n* = 3 (*p* < 0.05).

**Figure 5:**
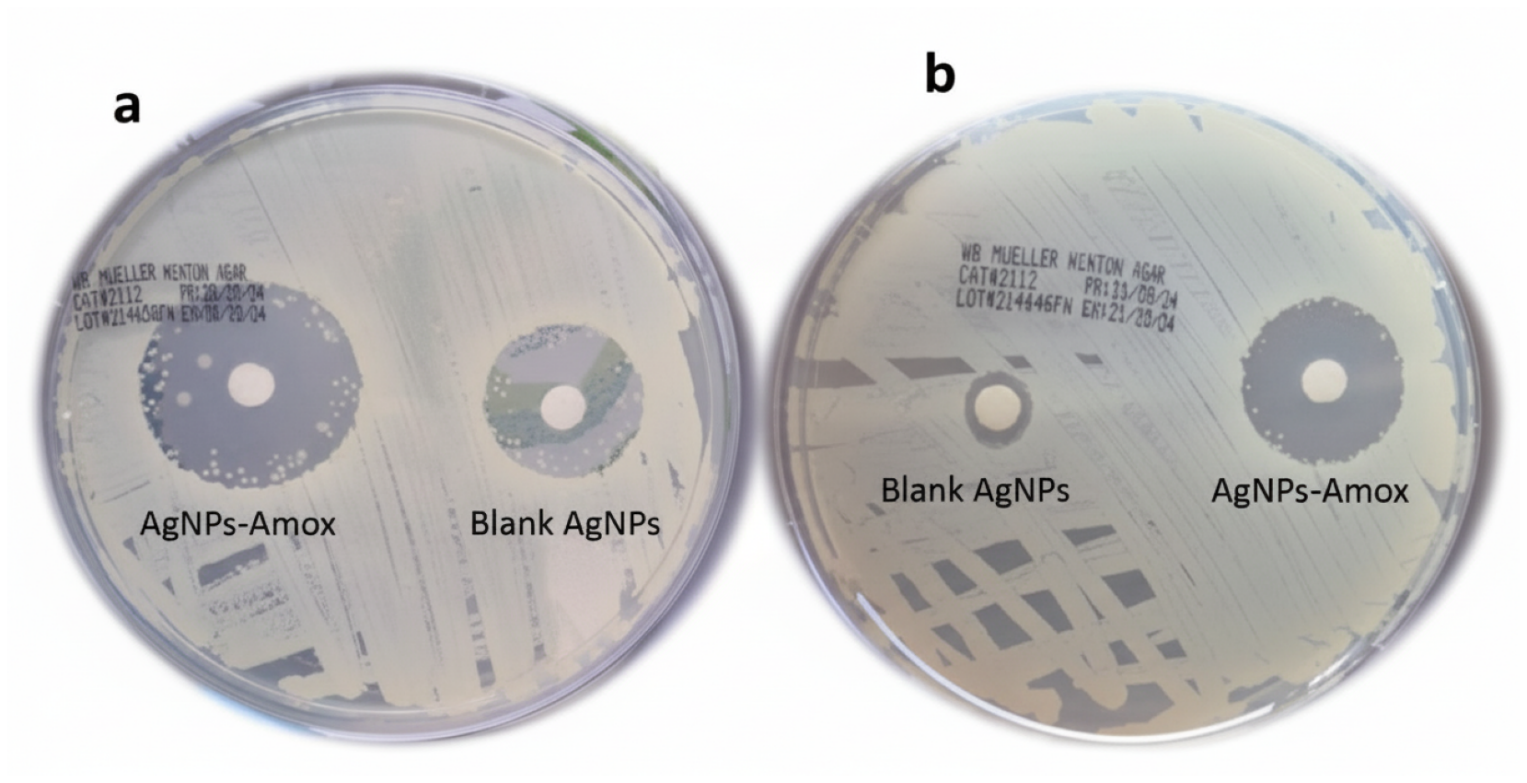
Kirby-Bauer antibiotic susceptibility test results showing zones of inhibition for (A) *S. aureus*; AgNPs-Amox 2.2 mm, blank AgNPs 1.4 mm, and (B) MRSA; AgNPs-Amox 1.6 mm, blank AgNPs 0.1 mm. Both strains were treated with AgNPs-Amox at 2. High bacterial susceptibility; blank AgNPs showed minimal activity.

The antimicrobial efficacy of AgNPs-Amox was assessed against MRSA and *S. aureus* using growth assays and antibiotic susceptibility testing. The minimum inhibitory concentration was established at two mg/ml for both bacterial strains. Growth curves demonstrated significant bacterial growth suppression upon treatment with AgNPs-Amox at this concentration compared to the untreated controls (Fig. 4). The Kirby-Bauer disk diffusion assay further corroborated these findings, showing pronounced zones of inhibition for both MRSA and *S. aureus* when exposed to AgNPs-Amox at two mg/ml (Fig. 5). By contrast, the blank AgNPs exhibited minimal antibacterial activity, indicating the critical role of the conjugated antibiotic in antimicrobial.

**Table 1:** The particle size, PDI and *ζ*-potential.

Average	AgNPs -Amox	AgNPs
Z average diameter (nm) ± SD	230 nm ± 2.1	120 nm ± 2.1
Polydispersity Index (PDI) ± SD	0.31 ± 0.005	0.21 ± 0.005
Zeta potential (Mv) ± SD	−32 mV ± 0.5	−24 mV ± 1.50

### Anti-virulence and biofilm inhibition

**Figure 6:**
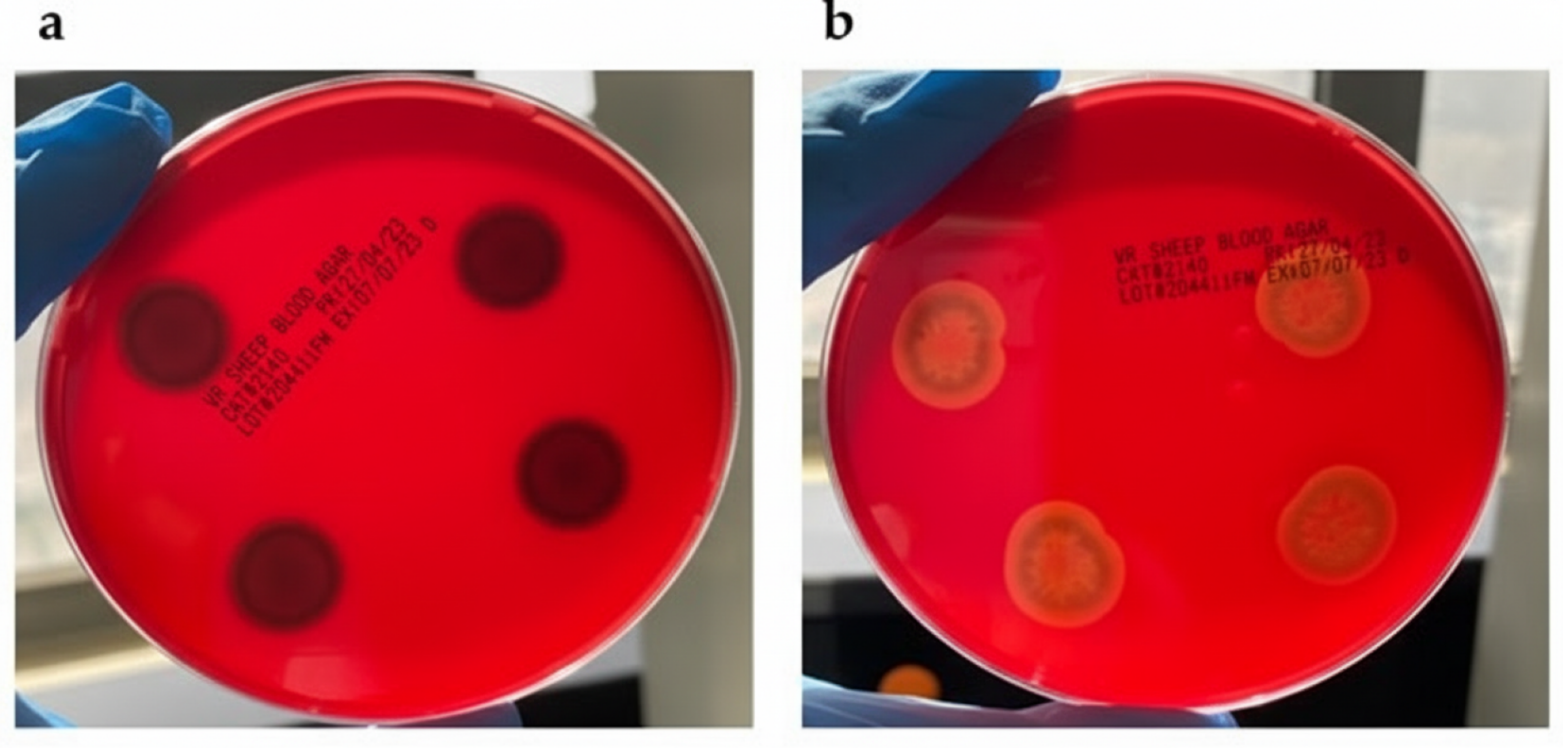
Hemolytic activity assay against MRSA: (A) complete suppression of hemolysis by AgNPs-Amox at MIC (2 mg/ml), zone diameter is 0 mm (B) partial suppression by blank AgNPs at same concentration from the diameter zone were 0.2 mm to o.1 mm.

The ability of AgNPs-Amox to mitigate virulence factors was investigated by measuring hemolytic activity and biofilm formation. Hemolysis is considered absent when no clear zone forms around the colony, and present when a clear zone of lysis is visible. At the MIC concentration two mg/ml, AgNPs-Amox completely suppressed hemolysis induced by MRSA, whereas blank AgNPs only partially inhibited this activity (Fig. 6).

In biofilm inhibition assays, the percentage of biofilm eradication was calculated to untreated control samples, which were assigned 100% biofilm biomass. The biofilm biomass was quantified using the crystal violet staining. After staining, the dye bound to the biofilm was solubilized, and absorbance was measured at 590 nm. The percent eradication was then calculated as:

% Eradication = [(OD of control – OD of treated)/OD of control] ×100.

**Figure 7:**
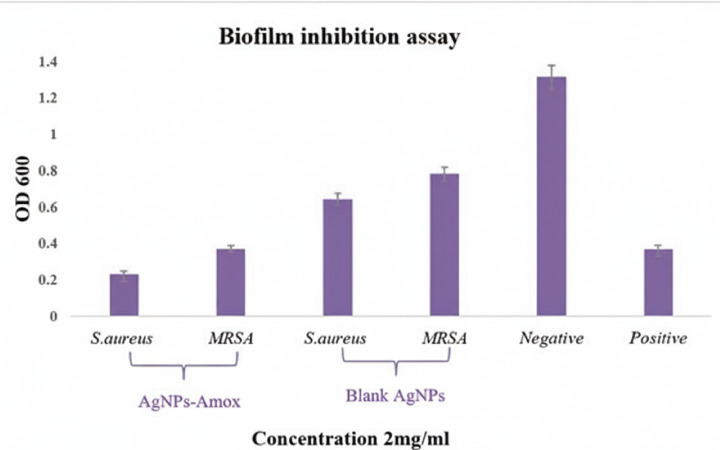
Biofilm inhibition assay comparing AgNPs-Amox and blank AgNPs effects on bacterial biofilm formation. AgNPs-Amox achieved 80% inhibition *versus* 25% by blank AgNPs at 2 mg/ml, negative control bacterial biofilm without treatment, positive control bacterial.

**Figure 8:**
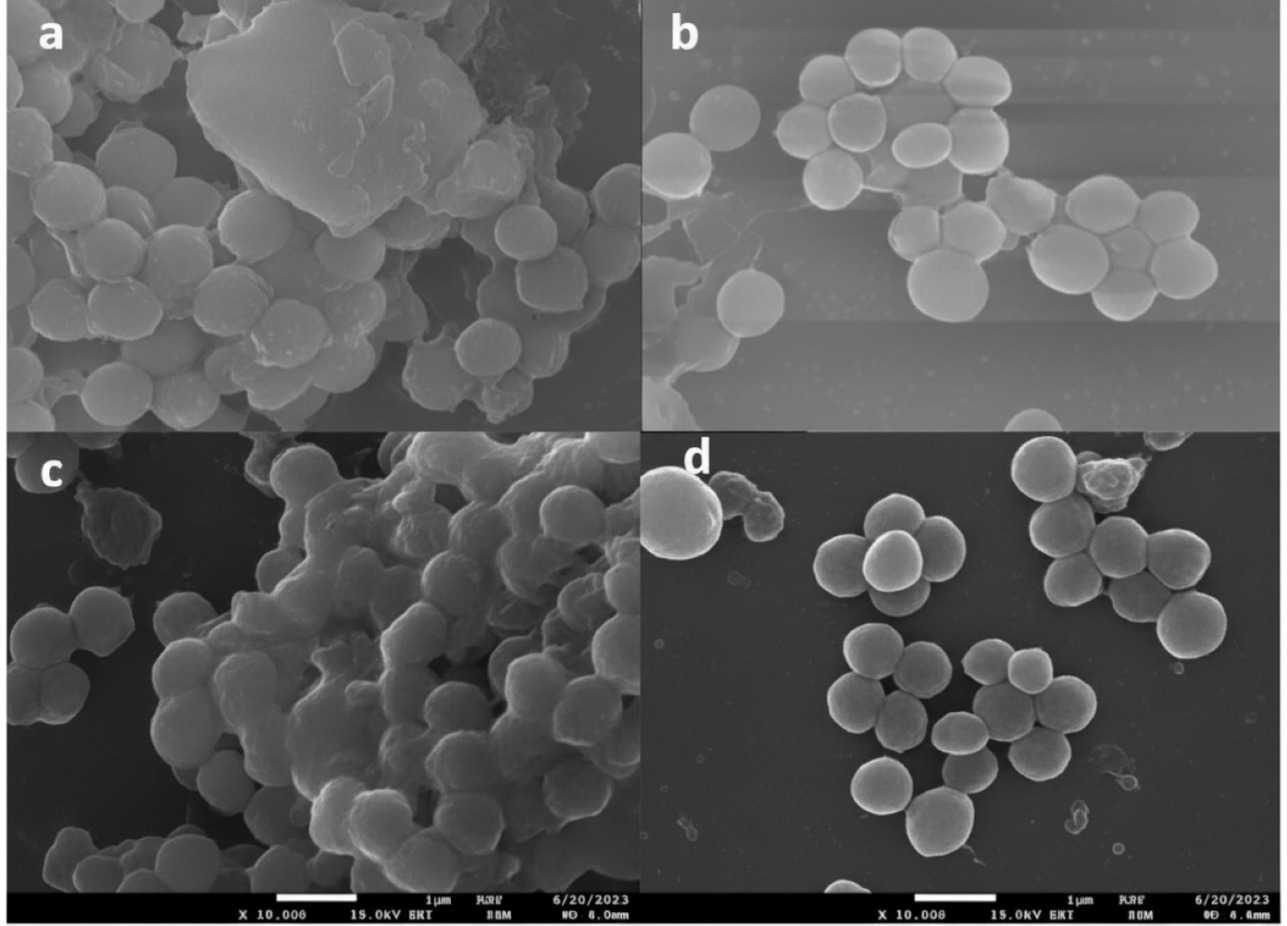
Scanning electron microscopy (SEM) images showing the effect of nanoparticles on biofilms: (A) untreated *S. aureus*, (B) *S. aureus* treated with AgNPs-Amox, (C) untreated MRSA, (d) MRSA treated with AgNPs-Amox.

AgNPs-Amox achieved an approximately 80% reduction in biofilm formation, significantly exceeding the 25% reduction observed with blank AgNPs at the same concentration (*p* < 0.05) (Fig. 7). Scanning electron microscopy (SEM) analyses revealed notable disruption of biofilm architecture in bacteria treated with AgNPs-Amox compared to untreated controls, further substantiating the potent antibiofilm effect of the formulated nanoparticles (Fig. 8).

### Cytotoxicity result

**Figure 9:**
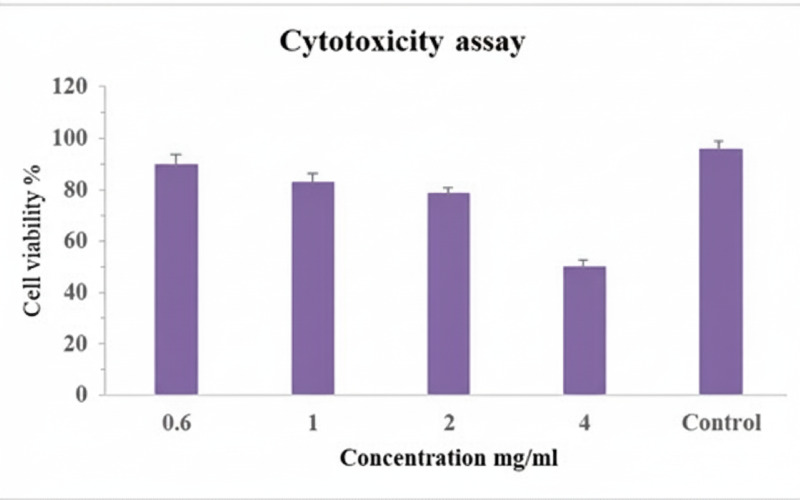
Cytotoxicity assay of AgNPs-Amox on endothelial cells. The higher concentration (4 mg/ml) were associated with reduced cell viability. MIC concentration demonstrating cytocompatibility. Data shown as mean ± SD, *n* = 3 (*p* < 0.05).

The cytotoxic potential of AgNPs-Amox was evaluated using endothelial cells exposed to increasing concentration of the nanoparticles, ranging from 0.5 mg/ml to four mg/ml. Results showed that higher concentration correlating with decreased cell viability. However, at the MIC concentration of two mg/ml, the treatment was found to be cytocompatible, demonstrating statistically significant maintenance of cell viability (*p* < 0.05) (Fig. 9). These findings suggest a favorable therapeutic window where antimicrobial efficacy is achieved without detrimental effects on endothelial cells.

## Discussion

The emergence of MRSA as a formidable pathogen within both healthcare and community environments presents a significant challenge to current antimicrobial therapies, underscoring the urgent need for novel treatment strategies (Hasanpour et al., 2023). This study demonstrates that silver nanoparticles conjugated with amoxicillin (AgNPs-Amox) exhibit robust antibacterial and anti-virulence activities against MRSA and *S. aureus*. The observed enhancement in efficacy likely arises from the interplay among silver nanoparticles, acacia extract, and amoxicillin, which collectively augment antimicrobial potency and disrupt bacterial pathogenic mechanisms (Saleh & Hassan, 2024).

The physicochemical characterization of AgNPs-Amox confirmed successful drug encapsulation, as evidenced by an increased average particle size of 230 nm compared to blank silver nanoparticles, alongside a reasonably low polydispersity index (PDI) of 0.31, indicating a relatively homogeneous nanoparticle population. The measured zeta potential of −32 mV suggests stable nanoparticle dispersion, which is critical for maintaining colloidal stability and ensuring effective delivery to bacterial targets. Transmission electron microscopy further substantiated the morphological integrity and size increase following amoxicillin loading. Such encapsulation not only protects the antibiotic from premature degradation but also improves its bioavailability and facilitating enhanced antibacterial action (Nielsen et al., 2014).

Antibacterial assays reaffirmed the superior efficacy of AgNPs-Amox compared to silver nanoparticles or amoxicillin alone. The minimum inhibitory concentration of two mg/ml against both MRSA and *S. aureus* highlights a potent growth inhibitory effect. This antibacterial enhancement is congruent with findings from Surwade et al. (2019), who reported synergistic interactions between silver nanoparticles and β-lactam antibiotics, where nanoparticles facilitate antibiotic penetration and disruption of bacterial defenses, particularly against drug-resistant strains. The Kirby-Bauer assay also demonstrated significant inhibition zones for AgNPs-Amox, showing that nanoparticles combined with antibiotics can restore or enhance susceptibility in resistant bacteria (Rai, Yadav & Gade, 2018; Do et al., 2023).

The anti-virulence properties of AgNPs-Amox were evidenced by complete suppression of MRSA hemolytic activity at MIC, an important virulence determinant contributing to immune evasion and tissue damage. Such inhibition is pivotal in attenuating bacterial pathogenicity beyond simple bactericidal effects. Furthermore, the formulation achieved approximately 80% reduction in biofilm formation, outperforming the 25% inhibition by blank nanoparticles alone. Given that biofilms confer enhanced resistance to antibiotics and shield bacteria from host immune responses, their disruption represents a critical therapeutic objective in combating persistent MRSA infections. The observed antibiofilm efficacy aligns with documented nanoparticle-mediated interference with biofilm matrix synthesis (Palau et al., 2023).

Mechanistically, the synergistic antibacterial effect can be attributed to the multifaceted actions of silver nanoparticles, acacia extract, and amoxicillin. Silver nanoparticles exert broad-spectrum antimicrobial effects by disrupting bacterial membranes, generating reactive oxygen species (ROS), and impeding DNA replication. Acacia extract, known for its antimicrobial phytochemicals, likely potentiates these effects by further compromising bacterial membrane integrity and metabolic functions. Importantly, amoxicillin, typically ineffective against MRSA alone due to resistance mechanisms, demonstrates restored efficacy when encapsulated within AgNPs, which may protect the drug from enzymatic degradation and promote enhanced intracellular delivery (Rai, Yadav & Gade, 2018; Ibraheem et al., 2022).

The present findings establish AgNPs-Amox as a promising antimicrobial formulation that combines nanoparticle technology and conventional antibiotics to overcome bacterial resistance and virulence. This multifunctional approach represents a significant advancement over traditional monotherapies, potentially reducing required antibiotic doses and mitigating resistance development. Future investigations should extend to *in vivo* models and clinical evaluations to validate therapeutic safety and efficacy. The integration of nanotechnology with classical antibiotics holds great promise to address the escalating global threat posed by multidrug-resistant pathogens such as MRSA.

## Conclusion and Future Perspectives

This study confirms that silver nanoparticles conjugated with amoxicillin (AgNPs-Amox) exhibit significant antibacterial and anti-virulence effects against MRSA and *S. aureus*. The capacity of AgNPs-Amox to inhibit key bacterial virulence factors such as hemolysis and biofilm formation underscores its potential not only to eradicate bacteria but also to attenuate their pathogenicity, an essential criterion for addressing persistent and chronic infections. Loading capacity and encapsulation efficiency were not determined in the present work, and this is acknowledged as a methodological limitation. The primary aim of this study was to evaluate the antimicrobial efficacy of the formulation rather than to provide a comprehensive pharmaceutical characterization. Future investigations will therefore focus on extensive physicochemical characterization, including quantitative assessment of loading capacity and encapsulation efficiency and long-term toxicity of AgNPs-Amox in suitable animal models. Additionally, the examination of the formulation’s efficacy against biofilm-associated infections *in vivo* will provide valuable insights into its therapeutic potential in complex physiological environments.

##  Supplemental Information

10.7717/peerj.20924/supp-1 Supplemental Information 1 Biofilm absorbance

## Additional Information and Declarations

### Data Availability

The following information was supplied regarding data availability: The raw data is available in the Supplemental File.
